# Modelling the Health Impact of an English Sugary Drinks Duty at National and Local Levels

**DOI:** 10.1371/journal.pone.0130770

**Published:** 2015-06-29

**Authors:** Brendan Collins, Simon Capewell, Martin O’Flaherty, Hannah Timpson, Abdul Razzaq, Sylvia Cheater, Robin Ireland, Helen Bromley

**Affiliations:** 1 University of Liverpool Management School, Liverpool, United Kingdom; 2 Division of Public Health, University of Liverpool, Liverpool, United Kingdom; 3 Centre for Public Health, Liverpool John Moores University, Liverpool, United Kingdom; 4 Trafford Council, Trafford, United Kingdom; 5 Health Equalities Group, Liverpool, United Kingdom; School of Public Health, Zhejiang University, CHINA

## Abstract

**Background:**

Increasing evidence associates excess refined sugar intakes with obesity, Type 2 diabetes and heart disease. Worryingly, the estimated volume of sugary drinks purchased in the UK has more than doubled between 1975 and 2007, from 510ml to 1140ml per person per week. We aimed to estimate the potential impact of a duty on sugar sweetened beverages (SSBs) at a local level in England, hypothesising that a duty could reduce obesity and related diseases.

**Methods and Findings:**

We modelled the potential impact of a 20% sugary drinks duty on local authorities in England between 2010 and 2030. We synthesised data obtained from the British National Diet and Nutrition Survey (NDNS), drinks manufacturers, Office for National Statistics, and from previous studies. This produced a modelled population of 41 million adults in 326 lower tier local authorities in England. This analysis suggests that a 20% SSB duty could result in approximately 2,400 fewer diabetes cases, 1,700 fewer stroke and coronary heart disease cases, 400 fewer cancer cases, and gain some 41,000 Quality Adjusted Life Years (QALYs) per year across England. The duty might have the biggest impact in urban areas with young populations.

**Conclusions:**

This study adds to the growing body of evidence suggesting health benefits for a duty on sugary drinks. It might also usefully provide results at an area level to inform local price interventions in England.

## Introduction

Approximately 2 billion people in the world are already obese or overweight.[1] In the UK there has been a continuous increase in the proportion of the population with a body mass index (BMI) in the overweight (BMI 25–29.9 kg/m^2^) and obesity (BMI > 30 kg/m^2^) ranges since the 1980s. Based on these past trends, future forecasts predict a continued rise in adult obesity prevalence, from 26% to 48% in men and from 26% to 43% in women, by 2030. However, some recent evidence has shown a possible plateauing in these trends.[2] Obesity levels in the UK thus resemble those in the USA 15 years earlier.[3]

A global review suggested that recent spend on obesity related diseases represented 0.7%- 2.8% of a country’s healthcare costs. Furthermore, healthcare costs in obese people were typically 30% higher than those with healthy weight.[4] In the UK in 2006/07, obesity and overweight were estimated to cost the NHS £5.1billion, representing around 5% of total NHS spending. The estimated total NHS cost of obesity and overweight for England in 2015 will be approximately is £15.4 billion, representing around 15% of the NHS budget.[5] In England, ‘*Healthy Lives*, *Healthy People*: *a call to action on obesity in England* (2011)[6] proposed a target of achieving a clear reduction in obesity prevalence in both children and adults by 2020. It also called for closer working with the food and drink industry (‘the Responsibility Deal’) to negotiate voluntary agreements aimed to reduce the nation’s calorie uptake by 5 billion kcalories per day.[6] That alone may not be sufficient.

One policy intervention with a potentially high impact [7] is a duty on sugar sweetened beverages (SSBs). SSBs include sodas, fruit drinks, sport and energy drinks, sweetened ice teas and other beverages that contain added caloric sweeteners. Survey data show that consumption of these drinks has progressively increased since 1988 in the UK [8] and in the USA [9], and that SSBs are consumed by teenagers in particular. SSBs are a potentially important source of “empty calories” with minimal nutritional value. Individuals with the highest consumption of SSBs may have a 26% greater risk of developing type 2 diabetes than those with the lowest SSB consumption.[10] People do not feel satiated (full) after consuming liquid calories as they do from solid food calories; the human body has evolved to assume that any liquid we consume is most likely to be water.[11] Furthermore, when people reduce their SSB intake, they do not compensate by getting more calories from food.[12] In fact SSB consumption has been associated with greater consumption of salty foods.[13] Therefore reducing SSB intake should reduce the total calories consumed across the population and thus reduce obesity rates over time.[14]

In 2013, the UK Faculty of Public Health (UKFPH) and UK Health Forum therefore called for a duty on sugar sweetened beverages [15] based on evidence from Briggs and others.[16] In 2013 Sustain, the alliance for better food and farming, called for a 20p per litre duty on sugary drinks, raising an estimated £1billion per year which could then be used to subsidise children’s healthy food and drink schemes.[17] Hypothecated (ring-fenced) duties like this are uncommon in the UK; examples include the TV license, congestion charges and motorway charges.

SSB duties have now been implemented in 33 US states (on average at a rate of 5%), as well as a 5% tax in France while Hungary, Norway, Denmark, Samoa and Mexico have also successfully established SSB duties. In May 2014 the British overseas territory of St Helena, which has a population of around 4,000, introduced a 75p per litre excise duty on high sugar carbonated drinks. The island is remote and largely dependent on supplies from one ship. It may thus be possible to crudely evaluate any future health benefits from the excise duty.

The present study followed an evidence review and qualitative studies conducted as part of a wider programme of work commissioned by directors of public health (DsPH) across the North West of England [18] which was used as evidence for the North West Directors of Public Health to call for an SSB duty in their Public Health Manifesto.[19] It can also be used as evidence when thinking about local interventions to reduce SSB consumption, such as not allowing SSBs in schools, or not selling SSBs in council leisure centres.

In this current study, we aimed to build on previous modelling work and produce a policy model to enable individual local authorities in England to estimate the local impact of a SSB duty as well as facilitate the possible discussion around sugary drinks.

## Methods

### The Policy Model

**Fig 1:**
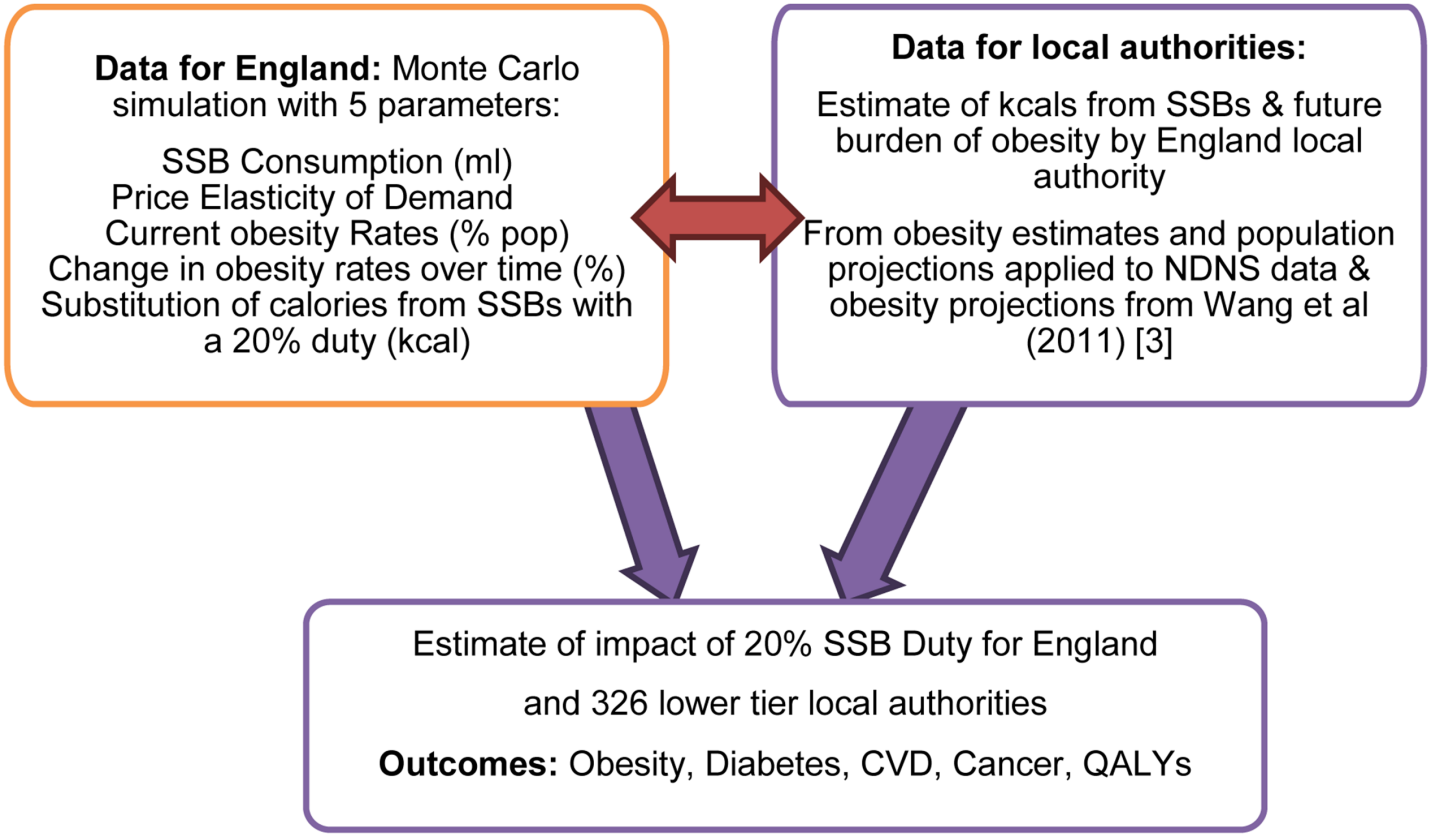
Schematic of SSB model for local authorities in England.

Fig 1 shows a schematic of the model. England and local authorities were used as these were populations for which obesity and disease projections were available. The outcomes were modelled for 41 million adults in England. The model involves applying a linear function to the estimated change in obesity-related diseases, based on the age-specific consumption data applied to the number of calories derived from SSBs, for each local authority. As a duty makes SSBs more expensive, so consumption reduces (elasticity). We assumed that elasticity was the same for all age and gender groups as we found no evidence for elasticity in particular demographic groups; there was evidence for differences in elasticity by income but the consumption data was not available by income groups. As the number of daily calories consumed reduces, so obesity rates fall. This then eventually leads to a decrease in obesity-related disease incidence and the QALY (quality adjusted life year) loss associated with these diseases.

### SSB Consumption

**Fig 2:**
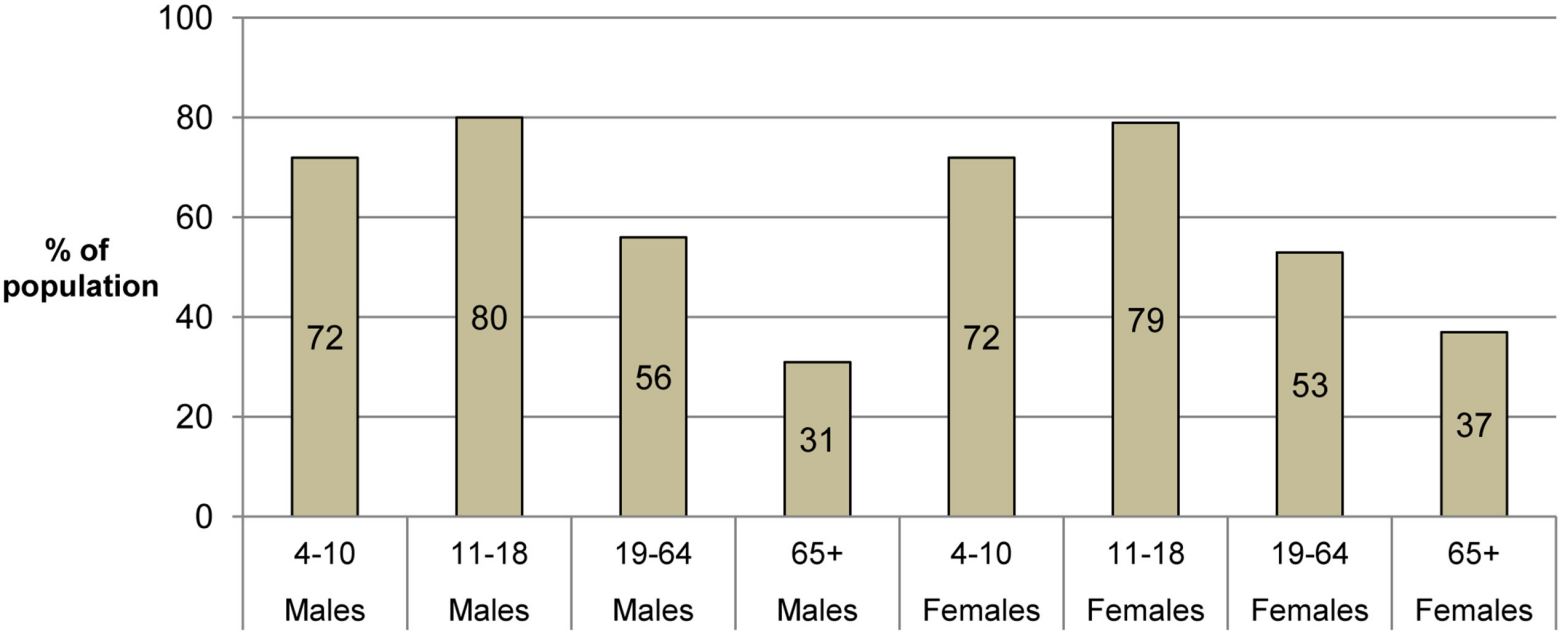
Percentage of England population who are SSB consumers by age group and gender. Data for England from NDNS 2008–10 [20].

Our main source for individual-level daily calorie intake data was the National Diet and Nutrition Survey (NDNS)[20] which has been carried out in England each year from April 2008. This also has data on the proportion of the population who consume sugary soft drinks, including the average number of grams per day consumed. We used the data for years 1–2 of the survey, from 2008–2010. Fig 2 shows the percentage of the population who are SSB consumers (in the NDNS it refers to ‘soft drinks, not low calorie’ which we have taken to be equivalent to our definition of SSBs—fruit juice is considered separately). The estimated average proportion of calories per day that came from SSBs varied between 0.8% in men aged 65+ to 4.9% in men aged 11–18. The biggest consumers are 11–18-year olds with around 80% being consumers. Men and women have a similar probability of being SSB consumers at ages 4–18. At 19–64 men are more likely to be consumers, whereas at 65 and over, women are more likely to be consumers.

**Fig 3:**
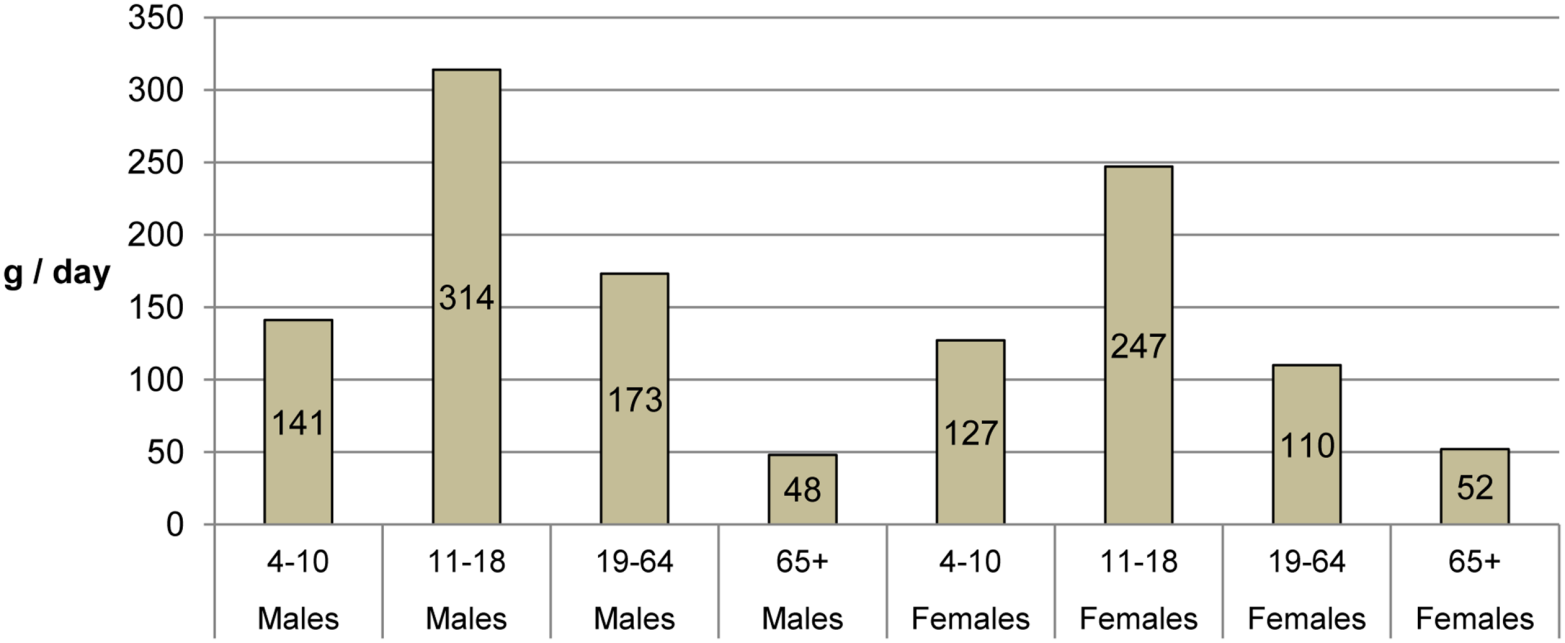
Average grams per day consumed of soft drinks by age group and gender (includes non-consumers of soft drinks). Data for England from NDNS, 2008–10 [20].

Fig 3 shows the average grams per day of soft drinks consumed, by age and gender. This includes people who do not consume any soft drinks at all, so their grams per day would be zero.

People in Britain consume an average of 145 ml each day of sugar sweetened drinks, based on the NDNS survey estimates [20] and 480 ml each day, based on manufacturer data (around 3–4 times as much).[21] This equates to between 50 and 170 kcal per day respectively. This difference could partly reflect wastage, and partly under-reporting in surveys. We therefore modelled a range of values based on these estimates. Table 1 shows the average consumption of SSBs and calories from SSBs from the NDNS and drinks manufacturer data, and the potential reduction in calories consumed as a result of a 20% duty.

**Table 1:** Average consumption per day of SSBs and impact of a 20% duty on calories (low and high estimates), data by gender and age group, based on NDNS data for 2008–10 [20].

Gender	Age group	Average g per day soft drinks from NDNS	Kcals per day soft drinks all (low estimate based on NDNS)	kcals per day increased by factor of 3.1 (478/156ml)based on manufacturer data (high estimate)	Example: Reduction in kcals per day from 20% duty resulting in 9.1% decrease in consumption	
					Low estimate (NDNS)	High estimate (Manufacturer data)
Male	4–10	141	44	136	4.00	12.41
Male	11–18	314	98	304	8.92	27.64
Male	19–64	173	54	167	4.91	15.23
Male	65+	48	15	46	1.36	4.22
Female	4–10	127	40	123	3.61	11.18
Female	11–18	247	77	239	7.01	21.74
Female	19–64	110	34	106	3.12	9.68
Female	65+	52	16	50	1.48	4.58

### Price Elasticity of Demand

For price elasticity, we used data from Ng et al.[8] which estimated the resultant reduction in number of millilitres of SSBs consumption for a 10% or 20% price increase. We then converted this to kcalories using the average number of kcalories per ml of SSB from the NDNS.

Ng et al. estimated that a 10% price increase would reduce SSB consumption by 4.6% and a 20% price increase would reduce consumption by 9.1% (a price elasticity of approximately -0.46). The cross price elasticity of low calorie soft drinks was very small, at -0.026 which indicates there is little substitution of high calorie drinks with low calorie drinks when prices increase so we have not factored this into the model (most low calorie drinks contain almost no calories anyway). We assumed that the same price elasticity applied across all age and gender groups. For England, we found that, averaged out across population groups, a 20% duty might produce a total reduction of 1.1 kcalories per day (low estimate) or 13.1 kcalories per day (high estimate) across the population.

### Impact of SSB Duty on Calorie Consumption and Health Outcomes

Wang et al.[3] presented a model of change in obesity-related outcomes over 20 years, and had a time series approach which took into account population change (Table 2). The number of older people is increasing, and older people are more likely to be obese, so this population change is a driver of obesity independent of other risk factors like SSB consumption. Wang et al [3] modelled the impact on diabetes, CHD & stroke, cancer, and change in QALYs resulting from a 20 kcalorie a day reduction in consumption, maintained over 3 years. We therefore have scaled this pro rata against the 1.1 kcalories per day (low estimate) or 13.1 kcalories per day (high estimate) reduction from a 20% SSB duty, although in the present study we are considering the impact of a permanent change in pricing policy rather than a 3 year intervention. If recent trends in obesity continue, by 2030 there will 545,000 additional cases of diabetes in the UK, and 2.2million additional QALYs lost. If a 20kcal reduction per person per day was achieved then average BMI would fall by 1% and there would be 179,000 fewer cases of diabetes and 3million QALYs gained.

**Table 2:** Outcomes associated with obesity scenarios, from Wang et al. Change in disease cases (incidence) and QALYs, 2010–2030, United Kingdom.

Disease	Scenario 1 –Assuming recent trends continue	Scenario 2 Assuming 1% reduction in BMI for every adult at baseline—needs average net reduction of 20kcal per person per day	Scenario 3 if obesity rates had remained at 1990 levels
Change in Diabetes cases	+545,000	-179,000	-897,000
Change in CHD and Stroke cases	+331,000	-122,000	-634,000
Change in Cancer cases	+87,000	-32,000	-177,000
QALYs gained or lost	-2,219,000	+3,011,000	+7,073,000

### Disease Cost Data

Table 3 shows the data sources for the annual costs of disease used in the model. Bowel cancer was chosen because it is one of the most common cancers related to obesity, and affects men and women. This cost estimate has also been used in other economic models around obesity and the impact on cancer [22–25].

**Table 3:** Annual cost of diseases used in model.

	Diabetes	CHD and Stroke	Cancer
Average cost per year of treatment	£1371	£4614	£8808
Details	Average healthcare cost of control group (usual treatment for type 2 diabetes).	Hospital cost of CVD event	Average cost per patient per year for bowel cancer
Source	Farmer (2009) [23]	NICE (2010) [24]	Trueman (2007) [25]

### Sensitivity Analyses

To account for uncertainty in the model parameters, we ran a Monte Carlo model simulation; in each, a random scenario was drawn from the data distributions for the parameters for England. The model was constructed in Microsoft Excel using VBA (Visual Basic for Applications). Each of the parameters was fitted to a normal distribution. 10,000 simulations were drawn from the data distributions for each parameter and the overall impact on average energy consumption was calculated for each simulation. This simulation generated 95% prediction intervals for these parameters. The parameters were assumed to be independent (i.e. not correlated). From this model a highest impact and lowest impact scenario were calculated by lining up the parameters together so that they affected the result in the same direction (a midway sensitivity analysis). The highest impact scenario assumed that the population SSB consumption was high, price elasticity of demand had a high negative value (i.e. consumers were sensitive to a change in price), and substitution of calories was negative. The lowest impact scenario assumed the converse. This England-level model then gave a set of kcal reduction factors that were applied to the population-specific data for each local authority.

## Results

**Fig 4:**
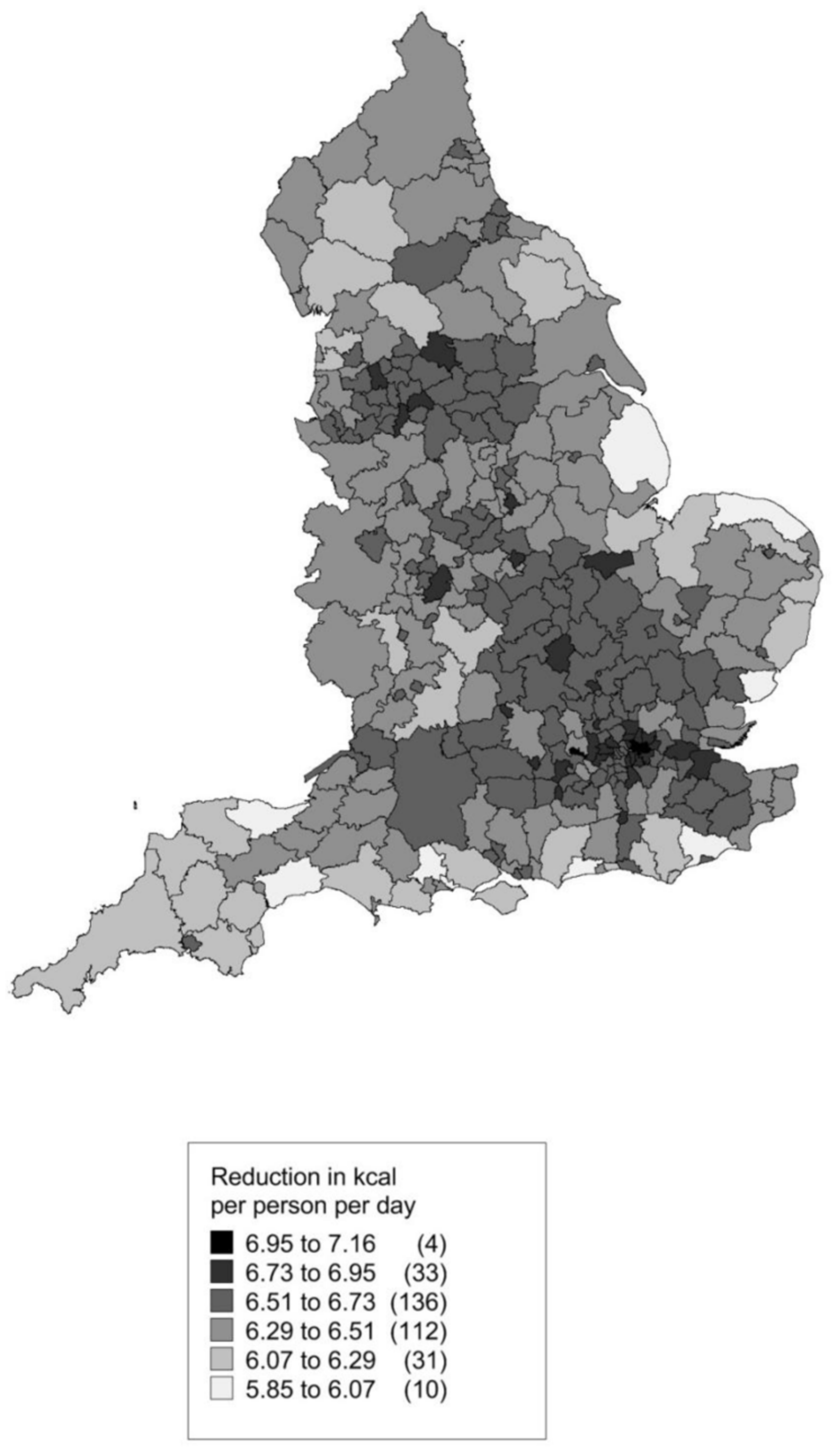
Thematic map showing estimated kcal reduction per person per day by local authority as a result of a 20% SSB duty.

**Fig 5:**
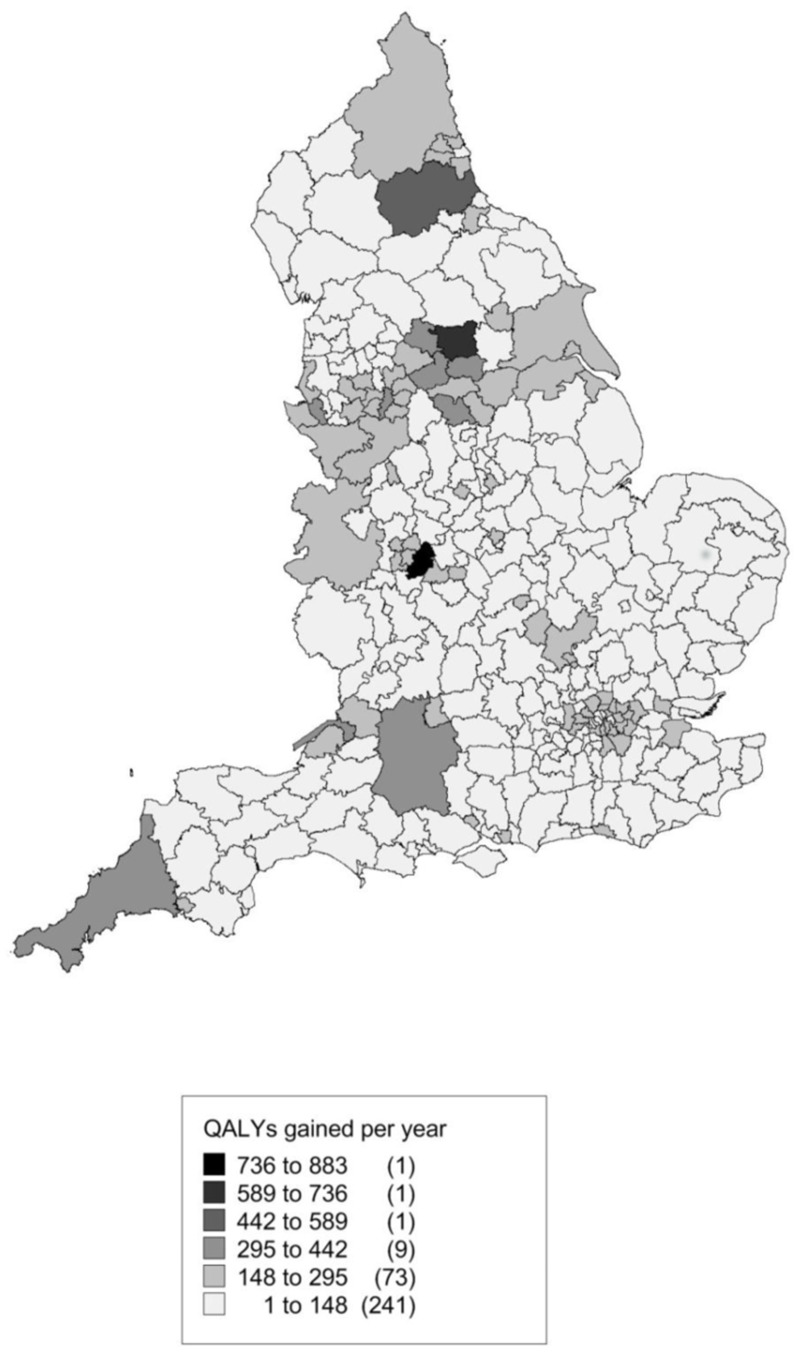
Thematic map showing estimated QALYs gained per year by local authority as a result of a 20% SSB duty.

Fig 4 shows a map of the modelled kcal reduction by local authority. Fig 5 shows the estimated QALYs gained. Both are driven by population size and age structure. The local authorities with the greatest number of QALYs potentially gained would be Birmingham, Leeds (two of the most populous local authorities in England) and Durham. Those with the smallest QALY expected gains would be three of the four local authorities with the smallest populations, namely the Isles of Scilly, City of London, and Rutland.

Table 4 shows the results from the Monte Carlo simulation for England. The overall impact for England is a reduction of between 1.1 and 13.1 kcalories per person per day (Table 4). The mean based on the simulation model was a reduction of 6.5kcal per person per day. The baseline obesity rates in the simulation model varied from 21.3% to 27.1% and the SSB consumption varied from 126ml to 473ml per day. The analysis suggested that a 20% SSB duty could result in approximately 2,400 fewer diabetes cases, 1,700 fewer stroke and coronary heart disease cases, 400 fewer cancer cases, and gain some 41,000 Quality Adjusted Life Years (QALYs) per year across England. Because we estimate that a duty would have a linear effect (within reasonable ranges), a higher duty of 30% would produce an effect of 1.5 times this, which would be around 3,600 fewer diabetes cases, 2,500 fewer stroke/CHD cases, 700 fewer cancer cases, and 61,000 QALYs gained.

**Table 4:** Results from Monte Carlo simulation model for England.

Parameter	Probability distribution for simulation (Standard Deviation)	Mean (based on simulations)	2.5% percentile—lowest impact scenario	97.5% percentile—highest impact scenario
SSB Consumption (ml/person/day)	Normal (91)	301	126	473
Price Elasticity of Demand	Normal (-0.12)	-0.691	-0.462	-0.922
Substitution of calories from SSBs with a 20% duty (kcal)	Normal (0.91)	1.78	0.03	3.57
Net impact—Calorie reduction per person per day	Calculated	6.5	1.1	13
Estimated change in diabetes cases/year	Calculated	-2432	-412	-4864
Estimated change in Stroke/CHD cases/year	Calculated	-1657	-280	-3314
Estimated change in Cancer cases/year	Calculated	-435	-74	-870
Estimated QALYs gained /year	Calculated	40,908	6,923	81,816
Total health cost savings per year (£)	Calculated	14,811,121	2,506,497	29,622,242

## Discussion

### Main Findings

Our study suggested that a 20% duty on sugary drinks could substantially reduce the burden of diabetes, cardiovascular disease and common cancers. There could be additional cash savings of approximately £15million from the reduction in healthcare costs for treating obesity related diseases. This is only considering the direct healthcare costs and not the human and productivity costs which would be much greater. In addition, the report by Briggs et al. predicted that a 20% duty would generate £276m in revenue in the UK, and the Sustain report estimated that a 20p per litre duty on SSBs could raise £1billion per annum in the UK.

The national target for reduction in calories in ‘*Healthy Lives*, *Healthy People*: *a call to action on obesity in England* (2011)[6] is for a 100 kcal reduction per person per day, so this reduction in SSB consumption would represent 1% to 13% of this reduction. Looking across England, the model suggests that the biggest change in calories might be for Newham (mid scenario 7.1 kcal per person per day) with the smallest expected change in West Somerset (5.9 kcals per person per day). This difference is driven by the age structure of the population. There is likely to be both an age effect, where younger people are more likely to be SSB consumers, and a cohort effect, that SSBs have become cheaper, are marketed more aggressively and have become more available over time, so that people born after 1980 are more likely to be SSB consumers throughout their lives than people born before 1980. This fits in with the insight work which found that older people saw consumption of SSBs as more of a treat whereas younger people often saw it as part of their daily routine. The duty might therefore generate the biggest benefits in urban areas with young populations and in areas with the greatest deprivation.

### Comparison with other Relevant Studies

Our results are based mainly on combining results from two models; the obesity modelled estimates produced by Eastern Region Public Health Observatory and the National Centre for Social Research, and the change in obesity related outcomes from Wang et al., with data from Office for National Statistics. Our overall results are reassuringly similar to those found in other studies, such as those by Briggs et al.[16], Tiffin et al.[26], and Sharma et al.[27] Briggs et al. predicted a 4 kcal per day reduction in energy consumption which, based on their cross sectional model, might lead to a 1.5% decrease in obesity rates (only slightly less than our predicted 6.57 kcal reduction). Sharma et al. compared a 20% flat rate sales tax (as in the present study) with a 20 cent/litre volumetric tax and found that the volumetric tax produced a greater weight loss across the population of SSB consumers.

### Strengths and Weaknesses of This Study

This study is based on high quality data and modelling studies, and it also estimates the impact at a local authority level. Local public health leaders can thus consider the potential health gains from a sugary drinks duty in their own area and compare it to other interventions. SSB consumption is highest in young people who may still be 10–30 years away from developing obesity-related diseases.[28]

This study has a number of limitations. Firstly, the model is dependent on the accuracy of other data and models. The parameter distributions used in the sensitivity analyses are rational but future research may throw up more robust empirical distributions. The current model did not factor in some potential predictive parameters like wastage; the WRAP report estimated soft drink wastage at around 7%.[29] Neither did we try to estimate the potential additional beneficial effect a tax might have on public perceptions; in France the 5% soft drinks tax has led to a drop in consumption of around 5%, which may be through a change in public attitudes rather than an economic effect on demand as a result of the price increase. This study also treated sugar calories as the same as any other calories; however, there is emerging evidence that calories from SSBs may have a greater effect than other calories in increasing risks of obesity, blood pressure, and coronary heart disease.[30] We also did not specifically address other important outcomes such as dental caries.[31]

Any duty on SSBs would probably be regressive, having a greater effect on people on lower incomes. However this negative effect might be trivial, amounting to an annual household penalty of barely £20.[27] And the harms might be further ameliorated if the revenue was spent on services targeting the most deprived areas such as through subsidised fruit and vegetables, or active travel. The Briggs [16] and Sharma studies [27] included price elasticity of demand estimates by income groups which could potentially be incorporated into this model in future if we had corresponding data for local authorities (or used income deprivation index as a proxy).

There is a risk that a duty may have a smaller effect than anticipated because the SSB producers or vendors absorb some of the cost of the duty; this depends on the producer surplus (profit). Sharma et al. [27] suggested that an excise duty on the volume of SSBs or the amount of sugar may have a greater impact in this case, such as the 20p per litre duty recommended by Sustain. The consumer surplus (the excess that people are willing to pay) is already factored into the price elasticity. However, in our preparatory insight work, some young people said that the price of the sugary drinks that they bought varied by so much that they were used to paying whatever price was charged.[18] There is also the issue of how to address 'meal deals' and other promotions where SSBs are currently included as options in other purchases. People might also switch to bulk buying or cheaper products. However, an excise duty based on volume or amount of sugar would mitigate this effect. This would also impact on fruit juices which were not included in the NDNS categories used in this study.

There has been a paradoxical effect observed over the last ten years where based on surveys, average calorie reduction has fallen while obesity prevalence has increased. Considering that an estimated 61% of adults are overweight or obese, the average numbers of calories per day seem lower than may be expected, given they are lower than the recommended number of calories per day for men of 2500 kcal and women of 2000 kcal to maintain a healthy weight. People in the survey may underestimate the number of calories they take in per day, for instance by underestimating calories from snacks or from alcoholic beverages.[32] These energy recommendations are based on moderate activity, whereas many people have a low level of activity. Underreporting is common in surveys like this and the NDNS has included doubly labelled water (DLW) measurements for a subset of participants to measure total energy expenditure and assess the extent of misreporting of energy intake. The DLW method involves participants drinking water labelled with deuterium and oxygen-18 which is then measured in urine samples to give an accurate indication of total energy expenditure (TEE). TEE is then compared with energy intake based on the food survey to illuminate where energy intake does not match energy expenditure. This NDNS study found that overall estimated energy intake was 27% higher than reported energy intake, or in other words, people were consuming about a quarter more calories than they reported on the food surveys.

### Implications for Policy & Future Research

More empirical research is needed on the effects of price on SSB consumption, particularly in young people, the biggest consumers, and also around ethnic minorities and deprived groups. People of Asian origin have an increased risk of diabetes and CVD at a lower BMI than other ethnic groups (27.5 kg/m^2^ instead of 30) so may be at higher risk of disease through SSB consumption. Energy drinks—many of which contain high amounts of caffeine and taurine—have been increasingly consumed over the last ten years.[33] Their effects have not been well researched.[34] Energy drink sales to young people are banned in Germany and Lithuania.

We recommend that local interventions around SSB price and availability should be piloted and evaluated. It would be interesting to look at the behavioural effect of price points, so for instance an increase from 95p to 99p for a drink may produce a smaller change in behaviour than from 99p to £1.02.

Currently around 30% of soft drinks consumed in the UK are diet drinks. The insight work brought out people's worries and some of the myths surrounding artificial sweeteners.[18] There are perceptions that artificial sweeteners, in particular aspartame, are bad for health and can cause cancer, which are mainly fuelled by discredited research.[35] In December 2013 the European Food Safety Authority clearly stated that aspartame was regarded as safe for all populations, including children and pregnant women, the only exception being individuals with phenylketonuria.[36] Might drinks with artificial sweeteners therefore represent the lesser of two evils?

In the future, data from the NDNS and from the Health Survey for England could perhaps be used to monitor SSB consumption and measure the impact of any fiscal measures to reduce SSB consumption.

## Conclusions

This study adds to the growing body of evidence quantifying the potential health benefits of a SSB duty and highlighting where the impact might be greatest. It might also be used to generate area level results to inform local discussions around price interventions.

## Supporting Information

S1 Table Results for individual local authorities in England. (XLSM)

S1 Text STROBE 2007 (v4) Statement—Checklist of items that should be included in reports of cross-sectional studies (DOCX)

## References

[1] Ng M, Fleming T, Robinson M, Thomson B, Graetz N, Margono C, et al Global, regional, and national prevalence of overweight and obesity in children and adults during 1980–2013: a systematic analysis for the Global Burden of Disease Study 2013. The Lancet 2014; 384(9945):766–781. 10.1016/S0140-6736(14)60460-8 24880830 PMC4624264

[2] Yanovski SZ, Yanovski JA. Obesity prevalence in the United States—up, down, or sideways?. New England Journal of Medicine 2011; 364(11):987–989. 10.1056/NEJMp1009229 21410367 PMC3345135

[3] Wang YC, McPherson K, Marsh T, Gortmaker SL, Brown M (2011). Health and economic burden of the projected obesity trends in the USA and the UK. The Lancet 2011; 378:815–25. 10.1016/S0140-6736(11)60814-3 21872750

[4] Withrow D, Alter DA. The economic burden of obesity worldwide: a systematic review of the direct costs of obesity. Obesity Reviews 2011; 12:131–141. 10.1111/j.1467-789X.2009.00712.x 20122135

[5] Scarborough P, Bhatnagar P, Wickramasinghe KK, Allender S, Foster C, & Raynor M. The economic burden of ill health due to diet, physical inactivity, smoking, alcohol and obesity in the UK: an update to 2006–07 NHS costs. J Public Health (Oxf). 2011 12;33(4):527–35. 10.1093/pubmed/fdr033 21562029

[6] HM Government. Healthy Lives, Healthy People: a call to action on obesity in England. 2011. Available: http://www.dh.gov.uk/en/Publicationsandstatistics/Publications/PublicationsPolicyAndGuidance/DH_130401 10.1017/S1368980019000053 PMC10260976 30838965

[7] Frieden TR, Dietz W, Collins J. Reducing Childhood Obesity through Policy Change: Acting Now to Prevent Obesity. Health Affairs 2010; 357–363. 10.1377/hlthaff.2010.0039, p. 358 20194973

[8] Ng SW, Mhurchu C, Jebb S, Popkin B. Patterns and trends of beverage consumption among children and adults in Great Britain, 1986–2009. Br J Nutr 2012; 108(3):536–551. 10.1017/S0007114511006465 22186747 PMC3310974

[9] Bleich SN, Wang YC, Wang Y, Gortmaker SL. Increasing consumption of sugar-sweetened beverages among US adults: 1988–1994 to 1999–2004. Am J Clin Nutr 2009; 89(1): 372–81. 10.3945/ajcn.2008.26883 19056548

[10] Malik VS, Popkin BM, Bray GA, Despres J-P, Willett WC, Hu FB. Sugar-sweetened beverages and risk of metabolic syndrome and type 2 diabetes: a meta-analysis. Diabetes Care 2010; 33:2477–83. 10.2337/dc10-1079 20693348 PMC2963518

[11] Pan A, Hu FB. Effects of carbohydrates on satiety: differences between liquid and solid food. Curr Opin Clin Nutr Metab Care. 7 2011;14(4):385–390. 10.1097/MCO.0b013e328346df36 21519237

[12] Berkey CS, Rockett HR, Field AE, Gillman MW, Colditz GA. Sugar-added beverages and adolescent weight change. Obes Res 2004; 12:778–88. 15166298 10.1038/oby.2004.94

[13] He FJ, Marrero NM, MacGregor GA. Salt intake is related to soft drink consumption in children and adolescents: A link to obesity? Hypertension 2008; 51:629–634. 10.1161/HYPERTENSIONAHA.107.100990 18287345

[14] Rada P, Avena NM, Hoebel BG. Daily bingeing on sugar repeatedly releases dopamine in the accumbens shell. Neuroscience 2005; 134:737–44. 15987666 10.1016/j.neuroscience.2005.04.043

[15] UK Faculty of Public Health. A duty on sugar sweetened beverages: A position statement. 2013. Available: http://www.fph.org.uk/uploads/Position%20statement%20-%20SSBs.pdf.

[16] Briggs ADM, Mytton OT, Kehlbacher A, Tiffin R, Raynor M, Scarborough P. Overall and income specific effect on prevalence of overweight and obesity of 20% sugar sweetened drink tax in UK: econometric and comparative risk assessment modelling study. BMJ 2013; 347, f6189 10.1136/bmj.f6189 24179043 PMC3814405

[17] Sustain. A Children’s Future Fund—How food duties could provide the money to protect children’s health and the world they grow up in. 2013. Available from: sustainweb.org/publications/info/263

[18] Timpson H, Lavin R, Hughes L. Exploring the Acceptability of a Tax on Sugar-Sweetened Beverages: Insight Work. Centre for Public Health, Liverpool John Moores University 2013.

[19] Bates G, Jones L, Timpson H, Begley E. Hardcastle K, Hughes K, et al. “Top Ten for Number Ten” A Public Health Manifesto from the North West Directors of Public Health. 2014 Available: http://phlive.org.uk/wp-content/uploads/Manifesto.pdf.

[20] National Diet and Nutrition Survey: Headline results from Years 1 and 2 (combined) of the rolling programme 2008–9–2009–10. 2011. Available: https://www.gov.uk/government/publications/national-diet-and-nutrition-survey-headline-results-from-years-1-and-2-combined-of-the-rolling-programme-2008-9-2009-10.

[21] British Soft Drinks Association 2012 BSDA UK Soft Drinks Report. 2012. Available: http://www.britishsoftdrinks.com/PDF/UK%20soft%20drinks%20report%202012.pdf.

[22] Trueman P, Haynes SM, Felicity Lyons G, Louise McCombie E, McQuigg MSA, Mongia S, et al (2010) Long‐term cost‐effectiveness of weight management in primary care. Int J Clin Pract, 64(6): 775–783. 10.1111/j.1742-1241.2010.02349.x 20353431

[23] Farmer AJ, Wade AN, French DP, Simon J, Yudkin P, Gray A, et al Blood glucose self-monitoring in type 2 diabetes: a randomised controlled trial. Health Technology Assessment 2009; 13(15), iii–iv. 10.3310/hta13150 19254484

[24] NICE Prevention of Cardiovascular Disease: Costing Report. 2010. Available: http://www.nice.org.uk/guidance/ph25/resources/ph25-prevention-of-cardiovascular-disease-costing-report2.

[25] Trueman P, Lowson K, Bending M, Ganderton M, Saxby R, Chaplin S, et al Bowel cancer services: Costs and benefits Report to the Department of Health. 2007 York and Sheffield: York Health Economics Consortium and the School of Health and Related Research (University of Sheffield).

[26] Tiffin R, Kehlbacher A, Salois M. The effects of a soft drink tax in the UK. Health Econ. 2015; 24: 583–600. 10.1002/hec.3046 24677314

[27] Sharma A, Hauck K, Hollingsworth B, Siciliani L. (2014) The effects of taxing sugar‐sweetened beverages across different income groups. Health Econ. 2014; 23:1159–1184, 10.1002/hec.3070 24895084

[28] Ambrosini GL, Oddy WH, Huang RC, Mori TA, Beilin LJ, (2013) Prospective associations between sugar-sweetened beverage intakes and cardiometabolic risk factors in adolescents. Am J Clin Nutr 2013; 98(2): 327–334. 10.3945/ajcn.112.051383 23719557 PMC3712546

[29] WRAP [Waste and Resources Action Programme] Final Report: Household Food and Drink Waste in the United Kingdom 2012. 2013. Available: http://www.wrap.org.uk/content/household-food-and-drink-waste-uk-2012.

[30] DiNicolantonio JJ, Lucan SC. The wrong white crystals: not salt but sugar as aetiological in hypertension and cardiometabolic disease. *Open Heart* 2014; 1(1), e000167 10.1136/openhrt-2014-000167 25717381 PMC4336865

[31] Marshall TA. JADA Continuing Education: Preventing dental caries associated with sugar-sweetened beverages. JADA 2013;144(10): 1148–1152 24080931 10.14219/jada.archive.2013.0033

[32] Livingstone MB, Black A. Markers of the validity of reported energy intake. Journal of Nutrition 2003; 133:S895–S920. 10.1093/jn/133.3.895S 12612176

[33] Brown H. Energy drinks are a booming business. Food Manufacture website. 2009 Available: http://www.foodmanufacture.co.uk/Business-News/Energy-drinks-are-a-booming-business.

[34] Seifert SM, Schaechter JL, Hershorin ER, Lipshultz SE. Health effects of energy drinks on children, adolescents, and young adults. Pediatrics 2011; 13(3):511–528. 10.1542/peds.2009-3592 PMC3065144 21321035

[35] Lofstedt RE. Risk communication, media amplification and the aspartame scare. Risk Management 2008; 10(4), 257–284.

[36] EFSA ANS Panel (EFSA Panel on Food Additives and Nutrient Sources added to food). Scientific Opinion on the re-evaluation of aspartame (E 951) as a food additive. EFSA Journal 2013; 11(12):3496, 263 10.2903/j.efsa.2013.3496

